# Diagnostics, Efficacy, and Safety of Immunomodulatory and Anti-Fibrotic Treatment for Interstitial Lung Disease Associated with Systemic Scleroderma (SSc-ILD)

**DOI:** 10.3390/diagnostics15172243

**Published:** 2025-09-04

**Authors:** Dawid Piecuch, Edyta Hanczyk, Katarzyna Zemsta, Michał Zwoliński, Szymon Kopciał, Joanna Jońska

**Affiliations:** 1 Faculty of Medicine, University of Radom, 26-600 Radom, Poland; edyta.hanczyk25@gmail.com (E.H.); k.zemsta99@gmail.com (K.Z.); michzal714586@gmail.com (M.Z.); szymon.kopcial@gmail.com (S.K.); 2 Faculty of Medical and Health Sciences, University of Radom, 26-600 Radom, Poland

**Keywords:** interstitial lung disease (ILD), systemic scleroderma (SSc), immunomodulatory treatment, nintedanib, tocilizumab, riociguat, mucophenolate mofetil (MMF), cyclophosphamide (CYC)

## Abstract

Systemic scleroderma (SSc) is an autoimmune disease characterized by excessive collagen production and progressive fibrosis. As the disease advances, vascular injury leads to fibrosis of the skin and internal organs, among which interstitial lung disease (ILD) carries the worst prognosis. Recent advances in biomarkers, imaging techniques, and innovative therapies offer hope for improving outcomes and quality of life in patients with SSc and ILD. To evaluate the usefulness of disease biomarkers and the efficacy and safety of immunomodulatory therapies in SSc-associated ILD (SSc-ILD), a literature review was conducted using the PubMed database for studies published mainly over the last 5 years. After applying inclusion criteria, 53 clinical studies were analyzed. Treating SSc-ILD remains challenging, with therapeutic strategies aiming to suppress inflammation and limit fibrosis progression. Clinical studies have demonstrated moderate to good efficacy of immunosuppressants such as cyclophosphamide (CYC) and mycophenolate mofetil (MMF), showing improvements in lung function parameters, such as forced vital capacity (FVC), and slowing disease progression. Additionally, biological agents such as nintedanib and tocilizumab have shown promising results—nintedanib in reducing the annual rate of FVC decline and tocilizumab in decreasing inflammatory biomarkers and stabilizing pulmonary function. However, despite these therapeutic advances, many studies had small sample sizes, heterogeneous patient populations, and varying inclusion criteria. Given the challenges in diagnostics and the critical need to evaluate the efficacy alongside the safety of immunomodulatory and anti-fibrotic therapies in systemic sclerosis-associated interstitial lung disease (SSc-ILD), there remains a strong demand for large, well-designed, multicenter trials with clearly defined patient cohorts to reliably assess the long-term outcomes of agents such as tocilizumab and nintedanib.

## 1. Introduction

Systemic scleroderma (SSc) is an autoimmune disease characterized by excessive collagen production and fibrosis process. During the progression of the disease, there is vascular damage, developing fibrosis of the skin and internal organs, leading to a number of pathological changes, among which interstitial lung disease (ILD) is the worst prognostically [1,2,3]. In clinical practice, patients with SSc can be divided into two subtypes, depending on the degree of skin involvement. The two subtypes are limited cutaneous SSc (lcSSc) and diffuse cutaneous SSc (dcSSc) [1,4]. ILD in the development of SSc is characterized by the presence of lung damage, involving inflammatory processes and fibrosis [1,2,3,5,6,7,8]. Patients often present with ILD symptoms, such as cough, dyspnea and hypoxia, which significantly affect their quality of life and chronic medical care [9]. The main causes of death in patients with SSc include ILD and pulmonary arterial hypertension (PAH) [2,3,6,9,10,11,12,13,14,15,16,17,18,19,20,21,22]. Analyses show that the presence of antitopoizomerase antibodies (ATA) influences a higher risk of developing clinically significant ILD [23,24]. Patients with high-risk features, and especially in the early phase of the disease, are an important target for early intervention. An important factor in the progression of SSc-ILD controlled patients is a decrease in forced vital capacity (FVC) [6,7,8,9,10,11,12,13,14,15,16,17,18,20,25,26,27]. Determinations of this parameter have important clinical value in determining the slowing of pulmonary fibrosis during immunomodulatory therapies [28]. Due to the importance of ILD in the prognosis and management of patients with SSc, research into the pathogenetic mechanisms of this disease and the identification of effective treatment strategies are crucial. The introduction of new biomarkers, imaging diagnostic techniques, and innovative therapies offer some hope for improving treatment outcomes and quality of life for patients with SSc and ILD. Treating patients with ILD is challenging, and the effectiveness of therapy is dependent on the stage of the disease [29,30,31]. Therapeutic approaches include immunosuppressants, corticosteroids, and disease-modifying drugs [29,30,31,32]. Immunomodulatory therapy modifies the immune system to reduce inflammation and immune-mediated damage. In SSc-ILD, it is used to suppress autoimmunity and prevent progression of fibrosis. Progressive SSc along with ILD requires immunosuppressive treatment, which, depending on the patient’s clinical condition, includes cyclophosphamide (CYC) [6,20,25] and mycophenolate mofetil (MMF) [6,17,18,33]. Although mycophenolate mofetil (MMF) has not been formally approved by regulatory agencies specifically for the treatment of systemic sclerosis-associated interstitial lung disease (SSc-ILD), it is widely recommended in clinical guidelines and used as a first-line therapy based on evidence from clinical trials. Immunomodulatory therapies aim to suppress autoimmune inflammation and are commonly used in SSc-ILD, with drugs such as tocilizumab [7,8,34,35] and riociguat [26,36,37]. Antifibrotic treatments, exemplified by the tyrosine kinase inhibitor nintedanib [6,9,10,11,12,13,14,15,16,17,38,39,40], target fibrogenic pathways, slowing the progression of pulmonary fibrosis. It should be noted that nintedanib is a drug that has been approved for the treatment of idiopathic pulmonary fibrosis, while studies are still underway in the context of the efficacy of tocilizumab or riocyguat in inhibiting inflammatory processes and pulmonary fibrosis [6,7,8,9,10,11,12,13,14,15,16,17,18,20,25,26]. A crucial prognostic factor is to determine the impact of MMF treatment at the beginning of therapy in patients who will receive nintedanib at a later phase [6,17]. The essence of immunomodulatory treatment is to determine the extent of its effectiveness and safety. So, with that being said, this review primarily focuses on evaluating the efficacy and safety of immunomodulatory and anti-fibrotic therapies for interstitial lung disease associated with systemic sclerosis (SSc-ILD), while also briefly addressing recent diagnostic approaches relevant to clinical management.

## 2. Materials and Methods

**Figure 1:**
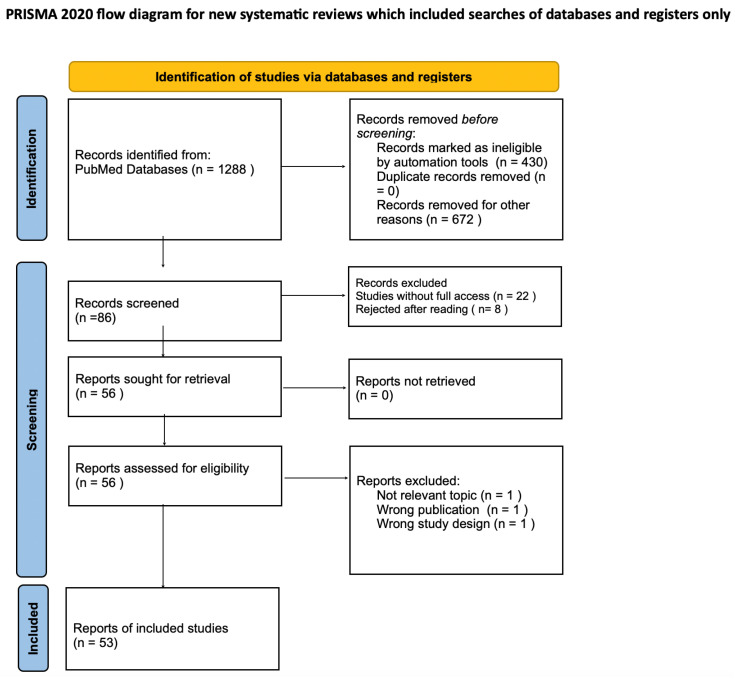
Preferred Reporting Items of Systematic Review and Meta-analyses (PRISMA) flow diagram.

A narrative review of the literature was conducted using the PubMed database in order to provide an overview of the diagnostics, efficacy, and safety of immunomodulatory and anti-fibrotic therapies for interstitial lung disease associated with systemic sclerosis (SSc-ILD). This research was conducted according to the PRISMA criteria and took place on 25 April 2025, and it was limited to publications from the past 5 years, reflecting the recent introduction and limited clinical experience with these treatment options. However, selected earlier studies of particular relevance had been also included to strengthen the contextual understanding. The search terms used were (Systemic Scleroderma) AND (Lungs). Only the following were included in the analysis: full-text, open-access articles classified as clinical trials, randomized controlled trials, meta-analyses, and books and documents. Records marked as ineligible by automation tools were excluded. After an initial search of the PubMed database, all records were selected by evaluating titles, abstracts, and full texts. Articles were excluded if they did not address the research question, represented an inappropriate type of publication (e.g., letters to the editor, comments, conference abstracts, case reports, reviews without meta-analysis), or had an inappropriate study design. Moreover, studies without access to the full text, not available in open access, or published in languages other than English were rejected. Five authors independently participated in the selection of studies and extraction of data. Each article identified in the search was initially screened by at least two reviewers based on title and abstract. Full texts of potentially relevant studies were subsequently evaluated for eligibility. Data were extracted using a standardized form, and discrepancies were resolved through discussion to ensure accuracy and cons. Figure 1 presents a flowchart of the study selection process, highlighting the articles that met the inclusion criteria.

## 3. Results

This review contains 53 studies. Table 1 summarizes the key characteristics of the included clinical studies, including therapeutic group, study drug, author, publication year, study design, methods, and main efficacy and safety outcomes. This review provides a basis for further discussion of clinical efficacy and safety, highlighting the diversity of study designs and patient populations.

### 3.1. Diagnostic and Prognostic Tools

The diagnosis and monitoring of interstitial lung disease associated with systemic sclerosis (SSc-ILD) requires a comprehensive approach that includes imaging, pulmonary function testing, and biomarker assessment. In addition to standard diagnostic tools such as high-resolution computed tomography (HRCT), FVC, and DLCO, emerging prognostic and predictive markers—such as metabolomic profiles, autoantibody patterns, and inflammatory mediators—may help identify patients at higher risk of disease progression and guide individualized treatment strategies.

Through the use of metabolomic techniques, it is possible to detect characteristic metabolic patterns that can not only support the early diagnosis of SSc-ILD but also serve as prognostic tools and predictors of response to immunosuppressive or antifibrotic therapy. However, it is important to emphasize that these metabolomic techniques are not yet widely available or routinely used in clinical practices, and their application remains largely within research settings. In the context of screening and diagnosis of interstitial lung disease associated with systemic sclerosis (SSc-ILD), it is important to refer to the latest guidelines of the American College of Rheumatology (ACR) and the American College of Chest Physicians (CHEST) from 2023. These guidelines recommend systematic screening of all patients diagnosed with systemic sclerosis for early detection and monitoring of interstitial changes in the lungs. High-resolution chest computed tomography (HRCT) and lung function assessment, including spirometry and carbon monoxide diffusion measurements (DLCO), are recommended as basic diagnostic tools. Monitoring should be performed regularly to enable early detection of disease progression and adjustment of therapy. Incorporating these guidelines into clinical practice increases the chances of improving the prognosis and quality of life of the patients.

The study by C. Meier et al. shows that the most effective differentiation between healthy subjects and SSc patients was obtained based on differences in l-tyrosine and l-tryptophan amino acid levels. In contrast, differentiation of patients with SSc-ILD from those without interstitial lesions was based mainly on abnormalities in the metabolism of l-threonine, xanthosine, 3-aminoisobutyric acid, and adenosine monophosphate. In contrast, patients with progressive ILD showed different metabolic profiles than those with the stable form, particularly in the concentrations of l-leucine, l-isoleucine, xanthosine, and adenosine monophosphate. In SSc-ILD, levels of L-leucine and xanthosine were associated with deterioration in lung function parameters such as FVC%. Xanthosine additionally correlated with lower DLco% and higher ILD-mortality prediction score (GAP index). Enzymatic tests confirmed that L-leucine and L-isoleucine help distinguish patient subgroups and are associated with lung function and prognosis. Thus, these metabolites have potential as biomarkers to assess the presence and severity of ILD in patients with SSc [3]. Despite these promising findings, the clinical use of metabolomic techniques to guide targeted therapies, such as tocilizumab (anti-IL-6), remains limited and mostly investigational. Current evidence does not yet support routine clinical application of metabolomics for treatment decisions, and further validation and standardization are required [8]. The study by Nihtyanova et al. included more than 1300 patients with various forms of SSc. The best 20-year survival (65.3%) and the lowest rates of pulmonary fibrosis (8.5%), renal crisis (0.3%), and cardiac involvement (4.9%) were found in patients with limited cutaneous SSc (lcSSc) and positive anti-centromere antibodies. The frequency of pulmonary hypertension (PH) in this group was similar to the average of the general population. In contrast, lcSSc patients with anti-Scl-70 antibodies and those with diffuse SSc (dcSSc) had the highest risk of pulmonary fibrosis (86.1% and 84% at 15 years, respectively). Patients with dcSSc and anti-Scl-70 showed the worst 20-year survival (32.4%) and high incidence of cardiac involvement (12.9%). Patients with lcSSc and these antibodies were less likely to have other complications and had the lowest incidence of pulmonary hypertension (6.9%) and the second highest survival rate (61.8%). The highest incidence of renal crisis (28.1%) was recorded in patients with anti-polymerase RNA antibodies. The anti-U3 RNP group had the highest rates of pulmonary hypertension (33.8%) and cardiac involvement (13.2%). Patients with lcSSc with other autoantibodies had a low risk of renal crisis and cardiac involvement (2.7% and 2.4%), and their outcomes were similar to those of the general population. In contrast, patients with dcSSc and other autoantibodies had a worse prognosis, with poor survival (33.6%) and multiple organ complications [23]. In patients with dcSSc-ILD, male gender and elevated CRP levels are important predictors of rapid lung function decline. Men treated with placebo had a higher risk of FVC decline of at least 10%, and the rate of decline was faster than in women. In the SMART cohort, male gender, the presence of ATA antibodies and a low initial FVC were associated with an FVC decline of less than 70%. Tocilizumab reduced the risk of worsening in patients with shorter disease duration, low IL-6 levels, and the presence of ATA. Although IL-6 correlates with disease severity, it does not clearly predict response to treatment. The results underscore the importance of early identification of high-risk patients for effective therapy [24]. Periostin, COMP, or Pro-C3 correlate with skin thickening as measured by mRSS, and SP-D is correlated with FVC, allowing early detection of organ involvement. They are useful in distinguishing clinical phenotypes. Differences in marker levels (e.g., SP-D or CXCL13) may reflect different pathophysiological mechanisms and degree of tissue involvement (lung vs. skin). They are also used to assess inflammatory and fibrotic activity. Elevated levels of CRP, IL-6, CRPM, or CCL18 indicate active inflammation, which may precede visible clinical symptoms. Biomarkers are being studied as indicators of the efficacy of therapies, especially targeted drugs, an example being tocilizumab (anti-IL-6) [8].

The diagnosis and monitoring of interstitial lung disease associated with SSc-ILD relies on a comprehensive diagnostic approach, including both imaging and functional tests. Because symptoms, such as a cough and dyspnea, may not appear until a more advanced stage of the disease, early diagnosis of SSc-ILD requires active search for lesions even in patients without respiratory symptoms. HRCT is the gold standard for diagnosis because it allows direct assessment of structural changes in the lung parenchyma. It allows detection of characteristic fibrous changes, such as reticularity (reticulation), thickening of interlobular septa, or a “honeycomb” pattern. It allows assessment of the extent and type of lung involvement (e.g., NSIP vs. UIP). Functional tests such as FVC and DLCO are also very important. FVC, when a reduced value is obtained, is indicative of restrictive respiratory failure, and DLCO assesses the gas exchange capacity of the alveoli [9,15,17,19,41,46,47]. The SENSCIS study showed that patients with SSc-ILD who reported a cough or dyspnea at baseline had a numerically greater extent of fibrosis on HRCT and lower FVC% and DLCO% compared to patients without these symptoms. However, even among patients without respiratory symptoms, significant changes were found in HRCT images (mean fibrosis extent > 30%) [9]. In a prospective study by Marialuis Bocchino et al. involving 83 patients with SSc, a computerized integrated index (CII) was developed that combines three basic densitometric measurements, mean lung attenuation (MLA), obliquity, and kurtosis, while eliminating redundant information using principal component analysis. During visual HRCT evaluation, interstitial lung disease was found in 47% of subjects. Patients with ILD had significantly worse CII values compared to those without lung involvement. Importantly, CII correlated strongly with lung function parameters both at baseline and during follow-up, as well as with immune and inflammatory markers such as serum levels of sIL-2Rα and CCL18. ROC analysis showed that the CII threshold value of 0.1966 allows us to distinguish patients with ILD with a sensitivity of 81% and specificity of 66% (AUC = 0.77). Interestingly, 34% of patients without visible ILD lesions on HRCT had CII below this value, and 67% of them had reduced lung diffusion capacity (DLco < 80% of predicted value) [19].

Arterial hypertension (AH) is a common complication in patients with systemic scleroderma and can affect the course of the disease and its complications. Among patients with arterial hypertension (AH), there was a significantly higher incidence of esophageal involvement (*p* = 0.011), the occurrence of finger ulcers (*p* = 0.017) and a higher mortality rate (*p* = 0.019). Scleroderma-related renal crisis occurred in nine patients (8.3%). Chronic kidney disease (PChN) was more common in patients with AH, especially at stages 2 (*p* = 0.013) and 3 (*p* = 0.07). The most severe stages of PChN, stages 4 and 5, were observed only in hypertensive patients. In addition, those with AH were more likely to have elevated uric acid levels (*p* = 0.007) [22]. Diagnostic advances related to biomarkers and imaging were discussed based on selected high-quality publications cited in recent clinical trials and expert consensus, outside the formal inclusion criteria.

The main diagnostic tools and their clinical relevance in SSc-ILD are summarized in Table 2.

### 3.2. Drugs Used in Interstitial Lung Disease Associated with Systemic Scleroderma

#### 3.2.1. Nintedanib

Nintedanib is an intracellular tyrosine kinase inhibitor and is an approved treatment for idiopathic pulmonary fibrosis [10,48,49]. The mechanism of nintedanib is to inhibit critical pathways leading to fibrosis and inflammation processes in the lung [48,50]. It mainly affects the release of profibrotic mediators, fibroblast proliferation and migration, and extracellular matrix deposition [12].

Distler O et al. conducted a double-blind placebo-controlled study to evaluate the efficacy and safety of nintedanib in patients with ILD. The study included 576 patients affected by SSc who had a symptom other than Raynaud’s within the past seven years and who underwent a high-resolution computer tomography (HCRT) scan confirming the presence of fibrosis involving at least 10% of the lung tissue. Overall, 52% of patients had a diagnosis of diffuse systemic scleroderma, while 48% of patients were taking MMF at the beginning of the study. Half of the patients received at least one dose (150 mg) of nintedanib twice daily, while the other half received a placebo. After 52 weeks, the decrease in FVC and the evaluation of skin involvement compared to baseline using the modified Rodnan scale (mRSS) were assessed, and the St. George’s Respiratory Questionnaire (SGRQ) was completed by the patients. The difference in the adjusted annual rate of change of FVC between the two groups of patients was 41 mL per year (in nintedanib-treated patients it was 52.4 mL per year, while in placebo-treated patients it was 93.3 mL per year). The results obtained using mRSS were similar and did not differ significantly between the two groups [10]. Reported adverse reactions or serious adverse effects occurred with similar frequency in both groups. In the nintedanib-treated group, more severe events resulting in treatment discontinuation were more frequent (16.6% versus 8.7% in the placebo group). The most common adverse reaction reported during nintedanib treatment was diarrhea, which occurred in 75.7% of patients [10,11,50].

Further, studies evaluated the safety and tolerability of nintedanib. Seibold JR et al. emphasized that adverse effects were consistent across demographic subgroups, highlighting the importance of dose adjustments to minimize side effects. The researchers concluded that choosing the right dose of the drug is key to reduce side effects and make it easier for patients to continue therapy [11]. Additionally, Khanna D et al. reported that nintedanib was particularly effective in patients with risk factors for rapid progression, such as short SSc duration (<18 months from onset of symptoms other than Raynaud’s), elevated inflammatory markers, and extensive dermatitis at the start of the study. The effect of nintedanib in slowing FVC decline was more pronounced in patients with risk factors for rapid progression of SSc-ILD than in the general study population. The findings presented in the study suggest that the progression of pulmonary fibrosis in patients with SSc-ILD is slowed by nintedanib regardless of risk factors for progression. These findings align with previous analyses, confirming that nintedanib is a valuable therapeutic option for the treatment of SSc-ILD, demonstrating significant efficacy in slowing FVC decline and the progression of pulmonary fibrosis [12,17,51,52,53].

On the other hand, M.Kuwana et al. evaluated the efficacy and effectiveness of nintedanib depending on the topoisomerase I antibody (ATA) score. The effect of nintedanib on reducing the rate of FVC decline (mL/year) seemed to be more pronounced in patients with a negative ATA result than in those with a positive ATA result (57.2 mL/year versus 29.9 mL/year) [13]. Similarly, Volkmann ER et al. examined the effect of nintedanib treatment in patients with SSc-ILD who had a cough and dyspnea compared to those who did not have such symptoms. In the placebo-treated group of the SENSCIS trial, the rate of FVC decline was similar regardless of whether patients experienced a cough and dyspnea at the start of the trial. The effect of nintedanib on reducing the rate of FVC decline appeared to be more pronounced in patients who did not have a cough or dyspnea at the start of the study compared to those who did, but there was no statistically significant difference in the drug’s effect between the groups [9]. Nintedanib slows the progression of SSc-ILD [6,9,10,11,12,13,14,15,16,17].

Furthermore, Justin K. Lui et al. note a smaller 40.9 mL/year decrease in the adjusted 12-month FVC rate compared to patients receiving a placebo. However, the study did not observe that nintedanib had any effect on the cutaneous manifestations of SSc [6]. Studies analyzing differences in the course of SSc show that Asians have a more rapid deterioration of lung function and thus a higher rate of mortality from SSc-ILD.

Geographic and demographic factors also play a role in treatment outcomes. Arata Azuma et al. evaluated the rate of reduction in FVC decline (mL/year) in Asian and non-Asian patients of a different race. After one year of treatment, nintedanib significantly reduced FVC decline in both groups of patients; patients taking the placebo achieved a 66% greater decline in FVC (mL/year). Moreover, the absolute reduction in lung function associated with taking MFF was 29.1 mL/year less than in patients treated with only nintedanib. Therapy was associated with adverse events, the most common were diarrhea (*n* = 309), nausea (*n* = 130) and vomiting (*n* = 101). SAEs were reported in 2.3% more people of Asian descent. A study by Arata Azuma et al. shows that nintedanib treatment of Asian patients in particular may have therapeutic benefit in slowing the progression of SSc-ILD [14].

Baseline disease severity did not significantly influence treatment efficacy. Denton CP et al. conducted a study evaluating the effect of ILD severity on HRCT at the start of treatment and FVC values assessed after 12 months of treatment. The study showed that patients starting treatment with greater pulmonary fibrosis did not experience a greater percentage decrease in FVC (mL/year) after one year of treatment. Moreover, differences in the degree of lung fibrosis on HRCT did not reflect the rate of FVC decline over 52 weeks. The researchers suggest that the drug has a benefit in reducing the progression of pulmonary fibrosis, regardless of the baseline severity of SSc-ILD [15,17].

Maher TM et al. and Highland KB et al. both supported the long-term benefits of nintedanib in reducing ILD progression, with or without concurrent MMF use. Toby M Maher et al. show that 82 patients treated with nintedanib had reduced predictable FVC% (i.e., FVC: 5–10% *n* = 26, 10–15% *n* = 7). The annualized coefficient of uncertainty (HR) for an absolute reduction in lung function parameters of more than 5% versus placebo reached 83%, and for a reduction in FVC of more than 10%, the HR was 64% [16]. Analysis of the study Highland KB et al. provides information on the beneficial therapeutic effect of nintedanib in patients who had to take the drug for at least 6 months before inclusion in the study treatment period. 

The researchers’ findings suggest that in patients with SSc-ILD, a cutting-edge biologic drug such as nintedanib is applicable in reducing the rate of ILD progression and that the inclusion of MMF is a safe and effective treatment option. Therapy was associated with side effects (consisting of diarrhea, vomiting, nausea, a cough, headache and abdominal pain, weight gain, and urinary tract infections), and in 15 patients, SAEs required discontinuation of therapy [17].

In long-term treatment, nintedanib is a safe and well-tolerated drug in patients with SSc-ILD, while side effects appear to be treatable [54].

#### 3.2.2. Cyclophosphamide

Cyclophosphamide (CYC) in intravenous (IV), oral (PO), and combination forms is a recommended drug for the treatment of systemic scleroderma with lung involvement, reducing inflammation and inhibiting disease progression results in an increase in percent predicted FVC% [6,20,25,55].

Researchers, including Lui JK et al., point out the beneficial effect of immunomodulatory drugs, including CYC, in early treatment. Comparing baseline FVC% with predicted FVC% after one year of treatment in patients with different stages of SSc-ILD, it appears that patients with mild forms of SSc-ILD receive the greatest benefit from treatment [6]. Roth et al. and Khanna et al. suggest that patients with more severe SSc-ILD on HRCT experienced the greatest benefit from cyclophosphamide (CYC) treatment, compared to those with milder forms. These studies indicate that severity of disease may not always correlate with the degree of benefit from treatment, providing a more balanced perspective on the effects of CYC [46,47].

Bruni C et al. conducted numerous studies to compare the effects and side effects of IV and PO drug use. In two groups of patients (*n* = 302) taking the drug orally (*n* = 149) and intravenously (*n* = 153), the percent FVC%, percent lung gas diffusion capacity (DLCO%), mRSS%, ILD progression rate, and mRSS changes were evaluated after one year of treatment time. Analysis of the above study endpoints showed no clinically significant differences in drug efficacy depending on the route of administration. However, after one year, more side effects accompanying oral therapy had been observed. Most notably a decrease in white blood cell count, hemorrhagic cystitis, or alopecia. IV-CYC therapy was associated with a higher rate of serious adverse events (SAEs) and the need for oxygen support in 11 patients during a follow-up. In addition, the researchers found that patients taking less than 15 g of the total dose (IV-CYC and PO-CYC), compared to those patients who had taken greater or equal doses had comparable therapeutic effects. What is to be mentioned here is that 19.8% more patients taking the IV drug were using higher values of disease-modifying antirheumatic drugs (DMARDs) before the study [25].

Tashkin DP et al. conducted a two-year study of 148 patients taking PO-CYC. In the second year of treatment, patients received no treatment or placebo, 26% of those treated took adjunctive therapy with corticosteroids, and 6% were put on disease-modifying therapy (azathioprine, MMF, and CYC). In both the SLS I and II-CYC trials, participants exhibited similar baseline characteristics. After accounting for initial disease severity, no significant differences were found in the changes to predicted FVC% (*p* = 0.535) or predicted DLCO% (*p* = 0.172) between the two groups. Both groups showed notable improvement in predicted FVC% during the first 3 to 12 months of CYC treatment, but no further improvements were observed thereafter. Additionally, CYC had no impact on DLCO in either group [20]. In addition, Tashkin et al. showed that in the second year of the Scleroderma Lung Study (SLS) II (CYC vs. MMF), in which patients in the CYC group received a placebo, FVC% continued to increase from baseline [56].

Volkmann ER et al. observed numerous side effects among subjects, the most common of which were hematuria (*n* = 12), anemia (*n* = 17), or leukopenia (*n* = 49). Patients tolerated the therapies badly while experiencing SAEs, with 11% of subjects dying during the study. It should be noted that SAEs including death may be due to the significant age advancement and comorbidities of the study subjects. The study suggests a potential benefit of prolonged CYC therapy in the treatment of SSc-ILD [20].

#### 3.2.3. Mycophenolate Mofetil

Immunosuppressive treatment with mycophenolate mofetil (MMF) suppresses disease progression and may result in a modest improvement in lung ventilation function in SSc-ILD.

One year after the introduction of immunomodulatory treatment in the form of MMF, in 47% of patients with moderate-stage SSc-ILD defined as the threshold value for FVC%, Lui JK et al. noted an increase in FVC. The differences in lung function parameters were greater in 53% of patients with moderate SSc-ILD than in patients with mild SSc-ILD who did not receive the treatment. This suggests reasonable early implementation of MMF treatment in patients with moderate SSc-ILD, as they may benefit most from improvements in FVC parameters [6].

Highland KB et al. conducted a study that compared the efficacy and safety of the combination of MMF and nintedanib compared to patients taking nintedanib alone or MMF and a placebo. After one year of treatment with therapy combining both drugs, a 26.3 mL smaller decrease in the adjusted mean FVC fall rate was noted compared to patients taking placebo. The absolute predicted FEV decrease of 0.33 and more was 11% less in patients taking MMF + nintedanib than in those treated with a placebo + MMF. During MMF treatment, the most common side effects were diarrhea (106/139 MMF + nintedanib, 48/140 MMF + placebo), nausea (43/139 MMF + nintedanib, 23/140 MMF + placebo), and skin ulcers (22/139 MMF + nintedanib, 23/140 MMF + placebo), while SAEs occurred in 26% of subjects taking MMF + nintedanib and 16% of subjects taking MMF + placebo. The study by Highland et al. was not a stand-alone “study,” but in fact involved a post hoc analysis of a subpopulation of Senscis study participants who were taking baseline therapy with MMF. The results indicated that nintedanib + MMF provided similar benefits over placebo + MMF that were found in the overall Senscis study population for nintedanib in monotherapy versus placebo. The combination of nintedanib + MMF was found to have a similar safety profile to nintedanib in monotherapy. It should be noted, however, that there was no evidence of additional benefit of nintedanib + MMF compared to nintedanib monotherapy [17].

The COVID-19 pandemic has highlighted the importance of evaluating the impact of SSc-ILD treatments on patients’ responses to vaccination. Researchers Sampaio-Barros PD et al. showed that patients treated with MMF had a lower seroconversion rate after vaccination with the inactivated SARS-CoV-2 vaccine. The seroconversion rate (SC) in SSc patients compared to the control group was 0.5 less after the first dose and 30.1 less after two doses of vaccination, respectively. MMF treatment was the only factor that had affected the decrease in antibody response in SSc patients. However, the study did not explore the relationship between drug dosage and response. Therefore, there is a great need to develop new treatments that guarantee a normal post-vaccination response, especially in patients with autoimmune rheumatic diseases (ARD) [18].

#### 3.2.4. Tocilizumab

Tocilizumab is a humanized IgG1 monoclonal antibody against the Il-6 receptor, registered for use in immune-mediated diseases. Previous analyses have indicated a possible beneficial effect of tocilizumab use in patients with early SSc-ILD [7,8]. In SSc, there is inflammation with increased levels of C-reactive protein (CRP), C-reactive protein metabolite (CRPM), and Interleukin 6 (IL-6).

Sheng XR et al. studied the mechanism of tocilizumab by analyzing serum biomarkers in healthy individuals and patients with dcSSc. They evaluated the pharmacodynamic effect and prognostic efficacy based on mRSS and FVC. They noted that inhibiting IL-6 receptors increased IL-6 levels, while pro-inflammatory macrophage activity decreased. The TGF-β-related pathway and COMP levels remained unchanged. Tocalizumab did not affect TGF-β activity and B cell-induced inflammation. This requires further research into their role in the pathogenesis of SSc. Treatment with tocilizumab affected changes in biomarkers related to macrophage activation, inflammation, and extracellular matrix transformation, including the processes of collagen neoepitope formation and degradation. Initial elevated levels of CRP, periostin, and SP-D showed prognostic tendencies related to more rapid deterioration of lung function, while higher baseline levels of IL-6, COMP, periostin, and Pro-C3 showed prognostic tendencies related to the severity of skin sclerosis. Biomarkers have the potential to be useful in drug development for SSc, but extensive, multicenter studies involving carefully defined patient groups are needed to fully assess their utility and reliability [8].

In a study conducted by Roofeh D. et al., the effect of treatment was evaluated and compared between a group of patients receiving tocilizumab (*n* = 104) and a group receiving a placebo (*n* = 106) based on the results obtained by HRCT and FVC measured by spirometry. From the entire group of patients included in the study, 136 of them were diagnosed with ILD. The researchers noted that in patients with dcSSc, there is a high incidence of ILD, with 77% of them developing lung involvement and having a moderate to severe form (lung involvement: sum of ground glass opacities, honeycomb and fibrotic reticulum > 10%). After 48 weeks of treatment, the mean % decrease in FVC was assessed, and it was noted that in tocilizumab-treated patients, it was −0.6%, compared to −4.0% in the placebo group, with *p* = 0.002. The mean change in the % pFVC in the TCZ arm was SD = 2, and in the PBO group, it was SD = 2.1. Additionally, the placebo group showed progression of pulmonary fibrosis on HRCT, and tocilizumab slowed the development of pulmonary fibrosis regardless of baseline quantitative lung involvement. Thus, it was hypothesized that tocilizumab may be a good therapeutic option by affecting the early phase of immunoinflammatory pulmonary fibrosis and resulting in preservation of lung function in patients with early-stage dc-SSc [7].

According to Hoffmann-Vold AM et al., every patient with SSc should be diagnosed to determine possible lung lesions with FVC and HRCT measurements, the results of which will allow early introduction of treatment [41].

#### 3.2.5. Rituximab

Rituximab is an anti-CD20 monoclonal antibody that reduces the number of B cells. In a study by Roofeh et al., rituximab treatment led to a significant improvement in FVC and mRSS compared to the control group over a 12-month period [29]. Available studies indicate that rituximab may have a beneficial effect on lung function in patients with SSc-ILD. A meta-analysis reported an average increase in FVC of 4.5% after 6 months of therapy and 7.0% after one year, as well as an improvement in DLCO of 3.5% and 4.1%, respectively. These changes translated into a clinically significant effect after only six months of treatment and were associated with a simultaneous decrease in mRSS. Another important aspect is the lower incidence of adverse events, especially infections, compared to patients receiving CYC or other immunosuppressive drugs. This phenomenon is likely due to the selective action of rituximab on B lymphocytes, without a significant effect on the T lymphocyte population [42,57]. The RECITAL study, a randomized, double-blind phase 2b trial, showed that rituximab does not demonstrate superiority over intravenous cyclophosphamide in the treatment of CTD-ILD. Both drugs led to similar improvements in FVC after 24 weeks of observation. However, RTX treatment was tolerated better, and the incidence of adverse events was lower compared to cyclophosphamide [43]. In the DESIRES study, beneficial clinical effects persisted after 24 weeks of rituximab therapy. Patients who received rituximab from the outset showed further improvement in mRSS, while patients initially treated with placebo and then rituximab also achieved a significant reduction in skin thickness after starting treatment. The treatment was well tolerated—serious adverse events occurred sporadically, and no patients died during follow-up [44]. Volkmann et al. observed stabilization of lung function parameters in most patients over 2 years of follow-up, with a good safety profile [30]. Ahmed et al. demonstrated a reduction in skin thickness and stabilization of lung function in patients with early-stage diffuse SSc [31]. In addition, the favorable safety profile of rituximab was confirmed, with infusion-related reactions being the most common adverse event and serious infections occurring rarely [33]. These data confirm that rituximab may be a potential therapeutic option for SSc-ILD, especially in patients with progressive disease despite first-line immunosuppression, although larger multicenter studies are needed.

#### 3.2.6. Pirfenidone

Pirfenidone, an antifibrotic drug, is being studied for the treatment of interstitial lung disease in systemic sclerosis. The Scleroderma Lung Study III evaluated combination therapy with pirfenidone and mycophenolate mofetil (MMF) compared to MMF alone. The results indicate that the combination of drugs accelerated the improvement in lung function (FVC) in the first 6 months of treatment and had a beneficial effect on patients’ quality of life. After 18 months, the differences were less pronounced. Combination therapy was associated with a higher incidence of adverse events, mainly gastrointestinal, but these were generally mild or moderate. Combination therapy with pirfenidone and MMF may be a promising treatment option for SSc-ILD, although further studies on long-term safety and efficacy are needed [58].

#### 3.2.7. Riociguat

Riociguat is a guanylyl cyclase stimulator. It has hypotensive effects and has found use in the treatment of pulmonary hypertension. Its inhibitory effects on vascular proliferation, inflammatory processes, and anti-fibrotic effects have been demonstrated on animal models.

Khanna D et al. conducted a study on its efficacy and safety in 121 patients with dcSSc. After 52 weeks, ILD patients saw a 2.7% decrease in mean FVC for riociguat users and a 7.6% decrease in the placebo group. Riociguat was well tolerated; however, more than 90% of patients experienced side effects and these were more frequent than in the placebo group. They mainly involved the gastrointestinal system (nausea and diarrhea), the nervous system (headache and dizziness), and the appearance of peripheral edema. In patients with a history of ILD, adverse events related to the respiratory system were reported with similar frequency in the riociguat-treated group and the placebo group. Based on measurements of pulmonary function in patients with ILD, researchers indicate its potential benefit and efficacy in this group.

Further research is needed on its use for treatment in patients with SSc-ILD [26] (Table 3).

## 4. Discussion

The use of metabolomics, HRCT, pulmonary function tests, and inflammatory and immunological biomarkers enables early diagnosis and monitoring of interstitial lung disease in systemic sclerosis (SSc-ILD) [3,8,9,15,17,19,22,24,41,46,47]. According to the 2023 guidelines of the American College of Rheumatology (ACR) and the American College of Chest Physicians (CHEST), mycophenolate mofetil is recommended as first-line therapy for the treatment of SSc-ILD. In addition, other therapeutic options are being considered, such as cyclophosphamide, rituximab, tocilizumab, nintedanib, and a combination of nintedanib and mycophenolate mofetil. These guidelines also emphasize the need for further research on the safety and efficacy of pirfenidone and its combination with mycophenolate mofetil in the treatment of SSc-ILD [45]. In addition, the 2024 American Thoracic Society (ATS) guidelines recommend the use of immunomodulatory and antifibrotic drugs in the treatment of all patients with SSc-ILD, regardless of disease progression. These guidelines recommend the use of mycophenolate mofetil and suggest considering other therapies, such as cyclophosphamide, rituximab, tocilizumab, nintedanib, and a combination of nintedanib and mycophenolate mofetil. In the case of pirfenidone, further research into its efficacy and safety in the treatment of SSc-ILD is recommended [59]. Metabolites such as L-leucine, isoleucine, xanthosine, and AMP show potential as biomarkers of disease progression and lung function [3]. HRCT remains the diagnostic gold standard, and the novel CII index allows objective assessment of lung involvement even in asymptomatic patients. ATA antibodies, cutaneous subtype (lcSSc vs. dcSSc), and male sex are strong prognostic factors [9,19,24]. Targeted therapies can slow ILD progression in patients with active inflammation. Biomarkers such as IL-6, CRP, SP-D, and CCL18 support phenotype differentiation and disease activity assessment [8]. Arterial hypertension worsens prognosis and increases the risk of chronic kidney disease, gastrointestinal involvement, and mortality [22]. Early identification of high-risk patients allows for more effective therapy and reduces the risk of organ complications.

Advances in the treatment of SSc-ILD represent a significant clinical challenge, mainly due to the heterogeneity of the disease course, the frequency of side effects, and the limited therapeutic efficacy of previously available drugs. An analysis of current literature reports indicates that both classic immunosuppressive drugs and novel targeted therapies can slow the progression of pulmonary fibrosis, although their efficacy and safety profile vary depending on disease progression, patient phenotype, and concurrent therapies. Nintedanib, a tyrosine kinase inhibitor, has demonstrated documented efficacy in slowing FVC decline in patients with SSc-ILD [10,12,17]. The SENSCIS trial (Distler et al.) confirmed its effect regardless of the presence of a risk factor for rapid progression, suggesting that nintedanib may be an effective drug in both early and more advanced stages of the disease [10,12]. At the same time, observed side effects, such as diarrhea (more than 75% of patients), underscore the need for appropriate dose selection and patient monitoring [11]. There are also interesting results from Asian populations, which indicate a more aggressive course of SSc-ILD and at the same time a better response to nintedanib treatment [14]. The variation in therapeutic response depending on ATA antibody status, the presence of dyspnea, and the degree of fibrosis confirms the clinical heterogeneity of SSc-ILD and the need to personalize therapy [9,13,15]. In contrast to nintedanib, CYC is a classic immunosuppressive drug whose efficacy has been confirmed in numerous studies, including SLS I and II [20,56]. CYC improves both FVC and slows disease progression during the first 12 months of treatment, with the effect not deepening over time, and long-term tolerability is limited due to frequent side effects such as leukopenia, hematuria, and anemia [20]. Analyses by Bruni et al. show that the route of administration (IV vs. PO) does not significantly affect efficacy, although the side effect profile may differ [25]. The results of these studies suggest that CYC may still be useful, especially in cases of more severe disease or as initial treatment [46,47]. MMF is now widely used as an alternative to CYC, especially due to its better tolerability. Its efficacy, documented in the SLS II trial, among others, suggests that MMF can lead to stabilization or even a slight improvement in lung function parameters, especially in patients with moderate SSc-ILD [6]. Moreover, a post hoc analysis from the SENSCIS trial showed no additional benefit from the combination of MMF with nintedanib relative to nintedanib monotherapy, suggesting that MMF may act mainly as a primary immunosuppressive treatment, to which antifibrotic treatment is possibly added [17]. However, it is worth noting the results regarding the post-vaccination response to COVID-19—patients treated with MMF show lower seroconversion, raising the question of the need to individualize treatment in the context of immune response [18]. Tocilizumab, as an IL-6 receptor inhibitor, appears particularly promising for the treatment of early forms of SSc-ILD, where inflammatory mechanisms play a predominant role. Studies by Roofeh et al. and Sheng XR et al. have shown that tocilizumab can significantly inhibit FVC decline and progression of fibrotic lesions in HRCT, regardless of their initial severity [7,8]. Despite the limited effect on TGF-β pathways, beneficial changes in biomarkers of inflammation and macrophage activity were observed. These findings suggest that anti-inflammatory treatment targeting IL-6 may have a role in early intervention before irreversible fibrotic changes occur. Riociguat, as a guanylyl cyclase stimulator, is still in the research stage, but early results suggest its potential efficacy in inhibiting FVC decline [26]. Due to its high incidence of side effects (>90% of patients) and limited data, its role in the treatment of SSc-ILD remains uncertain and requires further study. In conclusion, the current data indicate a beneficial effect of both immunosuppressive drugs (CYC, MMF) and antifibrotic (nintedanib) and biologic drugs (tocilizumab) in the treatment of SSc-ILD. However, these therapies differ in their mechanism of action, safety profile, and efficacy depending on the stage of the disease. Available data indicate that rituximab may have beneficial effects on lung function in patients with SSc-ILD. Meta-analyses have shown an increase in FVC of 4.5%, on average, after 6 months of therapy and 7% after one year, as well as an improvement in DLCO of 3.5% and 4.1%, respectively. These effects were clinically significant after only six months of treatment and were accompanied by a reduction in mRSS. Rituximab is also associated with a lower incidence of adverse events, including infections, compared to cyclophosphamide or other immunosuppressive drugs, due to its selective action on B lymphocytes with minimal effect on T lymphocytes. Clinical trials such as RECITAL and DESIRES confirm the efficacy and good tolerability of rituximab. Although it has not been shown to be superior to cyclophosphamide in improving FVC, the drug was better tolerated and associated with fewer adverse events. Long-term follow-up indicates sustained improvement in lung function and further reduction in skin thickness, with a low risk of serious complications [3,4,5,6,7,8,9,10,11,12,13,14,15,16,17,18,19,20,21,22,23,24,25,26,27,28,29,30,31,32,33,42,43,44]. Pirfenidone, an antifibrotic drug, is being studied in the treatment of SSc-ILD, especially in combination with mycophenolate mofetil. The Scleroderma Lung Study III showed that combination therapy accelerates FVC improvement in the first 6 months and has a beneficial effect on patients’ quality of life. After 18 months, the differences were less pronounced. The therapy was associated with a higher number of adverse events, mainly gastrointestinal, which were usually mild or moderate. These results suggest that pirfenidone in combination with MMF may be a promising treatment option for SSc-ILD, but further studies are needed to assess its safety and efficacy in the long term [58].

The use of appropriate therapy therefore requires careful evaluation of the patient’s phenotype, disease activity, and comorbidities. In the future, further comparative (head-to-head) studies, predictive biomarker analyses, and personalization of treatment based on clinical and immunological factors will be necessary. Particularly important seems to be the inclusion of studies on the impact of treatment on immune response and risk of infection, which may be crucial in the era of new health challenges, such as the COVID-19 pandemic.

## 5. Conclusions

Interstitial lung disease (ILD) is one of the most serious complications of systemic scleroderma (SSc) and represents a significant clinical challenge, leading to progressive loss of respiratory function and increased morbidity in patients. One of the most important clinical measures demonstrating the progression of ILD is FVC, which also determines the efficacy of the therapeutic therapies presented. HRCT of the lungs is a crucial tool for identifying characteristic interstitial changes, such as fibrosis and interstitial lung thickening. Treatment of ILD in SSc is challenging, with therapeutic strategies aimed at both controlling inflammatory processes and inhibiting excessive fibrosis. Corticosteroids, immunosuppressants, and disease-modifying drugs can be used depending on the individual patient’s clinical situation. Studies show significant efficacy of immunosuppressants, i.e., CYC, MMF, and biologic drugs, i.e., nintedanib, tocilizumab, and riociguat. In SLS I, CYC treatment resulted in greater benefit for FVC% in patients with the most severe form of SSc-ILD. The administration of CYC makes no difference to treatment efficacy; however, oral therapy can cause more side effects, the most common of which are leukopenia, hemorrhagic cystitis, or alopecia. IV-CYC was associated with higher rates of SAEs. During the one-year period, CYC treatment did not improve DLCO in either SLS I or SLS II, but an increase in FVC% was observed. A small percentage of patients may require treatment modification. Nevertheless, the need for CYC treatment in patients with SSc lesions cannot be clearly determined. Also, MMF slows down the progression of the disease and may cause a slight improvement in lung ventilation function. The inclusion of MMF in treatment is warranted in patients with mild SSc-ILD, but MMF has also been shown to be effective in patients with moderate to severe SSc-ILD. If patients with severe SSc-ILD do not respond well to MMF, the addition of an alternative therapy such as an anti-fibrotic drug or another immunomodulatory drug such as rituximab should be considered. MMF adds treatment efficacy benefits when combined with nintedanib. No additional primary therapy benefit was demonstrated with MMF in combination with nintedanib compared to nintedanib alone, although no adverse safety signal was demonstrated with the combination. Data indicate that rituximab may improve lung function and reduce skin thickness in patients with SSc-ILD, with a low risk of adverse effects, mainly due to its selective action on B lymphocytes. The RECITAL and DESIRES studies confirm its efficacy and good tolerability, although it does not surpass cyclophosphamide in improving FVC. Pirfenidone, used alone or in combination with mycophenolate mofetil, accelerates improvement in lung function and quality of life in patients in the initial months of therapy, with mild adverse effects. The results suggest that both drugs are promising therapeutic options in SSc-ILD, especially in patients with progressive disease.

Combination therapy is associated with side effects in the form of diarrhea, nausea, skin ulcers, and others. Among biologic drugs, nintedanib is approved in the treatment of idiopathic pulmonary fibrosis. The drug significantly reduced the corrected annual rate of change of FVC. The most common side effect reported during nintedanib treatment was diarrhea in 75.7% of patients; nausea and vomiting were also frequently reported. Therapy with nintedanib may need to be discontinued due to more severe side effects. The progression of pulmonary fibrosis in patients with SSc-ILD is slowed by nintedanib regardless of risk factors for progression, and more favorable outcomes are seen in patients who did not experience dyspnea and cough at the start of treatment. Nintedanib has the benefit of reducing the progression of pulmonary fibrosis, regardless of the baseline severity of SSc-ILD, and the combination with MMF is a safe and effective treatment model that can be applied in clinical practice. Studies have also presented a beneficial effect of tocilizumab in patients with early SSc-ILD. By inhibiting IL-6 receptors, the drug reduces pro-inflammatory macrophage activity, inflammation, and extracellular matrix remodeling. These results suggest that the drug may slow the process of lung fibrosis. Tocilizumab may be a good therapeutic option acting on the early phase of immunoinflammatory pulmonary fibrosis and causing preservation of lung function in patients with early-stage dc-SSc. The antiproliferative, antifibrotic, and anti-inflammatory effects of riociguat also reduce the progression of pulmonary fibrosis in the form of a smaller decline in FVC during treatment. Side effects include nausea, diarrhea, headache, and dizziness, and peripheral edema. There is a need for further larger, multicenter studies with carefully defined patient groups to fully assess the utility and safety of tocilizumab and nintedanib in SSc-ILD. Several promising agents are currently under investigation for SSc-ILD, including rituximab, abatacept, and lenabasum, which target distinct immunologic and fibrotic pathways. Combination strategies involving immunosuppressants and anti-fibrotic agents are being explored to address both inflammation and fibrosis simultaneously. Personalized treatment approaches based on biomarkers, genetic profiles, and disease phenotypes may improve therapeutic outcomes. Long-term multicenter randomized controlled trials with standardized diagnostic and outcome measures remain essential to establish optimal treatment algorithms for SSc-ILD.

## 6. Limitations of This Review

Limitations of this review are due to the availability of sources related to novel trials using biologic drugs in patients with ILD-SSc. The lack of uniform diagnostic criteria for ILD in SSc across studies may lead to variability in included cases, and the diversity of therapeutic approaches across studies may make it difficult to establish clear recommendations for optimal treatment of ILD in SSc. The literature search was conducted exclusively in the PubMed database. This limitation may result in the omission of publications available only in other databases. The analysis only included publications from the last five years, which was intended to reflect the latest state of knowledge on the subject. However, this approach may have resulted in the omission of older but still relevant studies that could have provided additional context and supplemented the obtained results.

## Figures and Tables

**Table 1:** Short study characteristics of main studies included for qualitative analysis.

Therapeutic Group	Type of Drug	Study	Year	*n*	Brief Description of Methods and Results	Summary of Results
Tyrosine kinase inhibitor	Nintedanib	Distler et al. [10] Seibold et al. [11] Khanna et al. [12] Kuwana et al. [13] Volkmann et al. [9] Lui et al. [6] Azuma et al. [14] Denton et al. [15] Maher et al. [16] Highland et al. [17]	2019 2020 2022 2022 2022 2020 2020 2023 2021 2021	*n* = 576 *n* = 576 *n* = 575 *n* = 576 *n* = 575 *n* = 566 *n* = 819 *n* = 576 *n* = 576 *n* = 576	Double-blind, placebo-controlled study with 576 patients (299 with diffuse SSc, 277 on MMF). Nintedanib reduced the decline in FVC (52.4 mL/year vs. 93.3 mL/year in placebo). No significant skin improvements on mRSS. Diarrhea was the most common adverse reaction (75.7%). Focused on safety and tolerability. Found consistent adverse effects across age, sex, race, and weight. Emphasized the importance of dose optimization. Studied effect on patients with rapid progression risk factors. Found greater FVC benefit in high-risk patients, but effects consistent regardless of risk factors. Assessed impact based on ATA status. Greater reduction in FVC decline in ATA-negative patients (57.2 mL/year vs. 29.9 mL/year). Analyzed cough/dyspnea effects. Greater FVC benefit in patients without initial symptoms, though differences were not statistically significant. Smaller decrease in 12-month FVC (40.9 mL/year). No effect on skin involvement. Compared Asian and non-Asian patients. Significant FVC decline reduction in both groups. Asians showed higher adverse event rates. Evaluated HRCT fibrosis severity at baseline vs. FVC after 12 months. Found no correlation between baseline severity and treatment benefit. Assessed HR for absolute FVC reductions > 5% and >10%. HR for >5% was 83%; for >10%, HR was 64%. Combined nintedanib with MMF. Adjusted FVC decline 26.3 mL/year less vs. MMF + placebo. Higher SAE rates but effective in reducing ILD progression.	Slows FVC decline and progression of pulmonary fibrosis. No significant skin benefits. Manageable side effects. Dose adjustment reduces side effects and improves patient adherence. Effective in slowing FVC decline in rapid progression cases and general population. Nintedanib may be more effective in ATA-negative patients. Reduces FVC decline regardless of cough/dyspnea symptoms. Preserves lung function, no impact on skin symptoms. Benefits Asian patients significantly; higher adverse events observed. Benefit independent of initial fibrosis severity. Predictable and substantial preservation of lung function. Effective in combination therapy; additional research needed for long-term effects.
Biological drugs	Tocilizumab	Sheng XR et al. [8] Roofeh et al. [29] Hoffmann-Vold et al. [41]	2023 2021 2022	*n* = 214 *n* = 556 *n* = 575	Investigated serum biomarkers. Tocilizumab inhibited macrophage activity but did not affect TGF-β pathways. Biomarkers such as CRP and IL-6 showed prognostic tendencies. Evaluated HRCT and FVC after 48 weeks. Tocilizumab reduced pulmonary fibrosis progression and preserved lung function. Highlighted early diagnosis of ILD for treatment initiation.	Potentially useful for early-stage immunoinflammatory fibrosis. Further multicenter studies needed. Effective in early-stage dc-SSc with lung involvement. Early detection and treatment crucial for better outcomes.
	Rituximab	Goswami RP et al. [42] Maher et al. [43] Ebata S et al. [44] Volkmann et al. [30] Ahmed et al. [31]	2021 2023 2021 2018 2022	*n* = 575 *n* = 145 *n* = 80 *n* = 148 *n* = 145	A meta-analysis of 20 studies (575 patients) showed that rituximab improved FVC and DLCO at 6 and 12 months, with a lower risk of infections compared to other immunosuppressive therapies. The RECITAL trial showed that rituximab was as effective as cyclophosphamide in improving FVC in CTD-ILD, but with fewer adverse events and lower steroid use. Favorable safety profile and no reported fatalities during the study period. Two-year study on oral CYC. FVC improved in the first year but plateaued in the second. Observed SAEs like hematuria, anemia, and leukopenia. Individualized treatment based on disease progression.	Rituximab improves lung function and skin thickness in SSc-ILD with low adverse effects, while pirfenidone, alone or with mycophenolate, quickly enhances lung function and quality of life; both appear promising for progressive disease.
	Riocyguat	Dinesh Khanna [26]	2020	*n* = 121	Assessed FVC in 121 patients after 52 weeks. FVC decline less in riociguat group (2.7%) vs. placebo (7.6%). High incidence of adverse effects.	Promising results for ILD; further studies needed for broader use.
Immunosuppressive drugs	Mycophenolate mofetil	Lui et al. [6] Highland et al. [17] Sampaio-Barros et al. [18]	2020 2021 2022	*n* = 576 *n* = 819 *n* = 204	Moderate-stage SSc-ILD patients saw FVC improvements after one year. Early implementation suggested greatest benefits. Combination with nintedanib showed less FVC decline vs. nintedanib or MMF alone. Common side effects included diarrhea and nausea. Assessed seroconversion rates after COVID-19 vaccination. MMF associated with reduced antibody response.	Effective in moderate SSc-ILD; early treatment recommended. Safe and effective combination therapy. Further research on long-term outcomes needed MMF decreases vaccine efficacy; further studies needed to optimize vaccination strategies.
	Cyclophosphamide	Bosch et al. [6] Bruni et al. [25] Volkmann et al. [20]	2020 2020 2019	*n* = 576 *n* = 302 *n* = 148	Compared FVC improvement across SSc-ILD stages. Mild cases benefitted most. Compared IV and PO administration. Found no major efficacy differences but higher side effects in oral group. Two-year study on oral CYC. FVC improved in the first year but plateaued in the second. Observed SAEs like hematuria, anemia, and leukopenia.	Effective in early-stage disease; moderate benefit in advanced stages. IV administration preferred due to lower side effects. Short-term efficacy observed; long-term benefit unclear.
	Rituximab	Roofeh et al. [29] Goswami R.P. et al. [42]	2021 2021	*n* = 556 *n* = 556	Evaluated HRCT and FVC after 48 weeks. Ritixumab reduced pulmonary fibrosis progression and preserved lung function.	Rrituximab treatment led to a significant improvement in FVC and mRSS compared to the control group over a 12-month period
Antifibrotic drugs	Pirfenidone	Dinesh Khanna [45]	2022	*n* = 51	The Scleroderma Lung Study III enrolled 51 patients to compare mycophenolate mofetil (MMF) alone versus MMF combined with pirfenidone in SSc-ILD.	Scleroderma Lung Study III with 51 patients showed faster FVC improvement during the first 6 months with MMF + pirfenidone therapy, with good treatment tolerability.

MMF—mycophenolate mofetil, FVC—forced vital capacity, mRSS—modified Rodnan skin score, HRCT—high-resolution computed tomography, ATA—anti-topoisomerase I antibody, ILD—interstitial lung disease, HR—hazard ratio, SAE—serious adverse event, TGF-β—transforming growth factor beta, CRP—C-reactive protein, IL-6—Interleukin 6, dc-SSc—diffuse cutaneous systemic sclerosis, PO—Per Os (oral administration), IV—intravenous, CYC—cyclophosphamide, SAEs—serious adverse events.

**Table 2:** Diagnosis tools and markers for SSc-ILD.

Tool/Marker Type	Method	Application	Examples/Indicators	Reference
Diagnostic tools	HRCT (High-Resolution Computed Tomography)	Gold standard for structural lung assessment	Reticulation, honeycombing, NSIP vs. UIP patterns, fibrosis extent	Elizabeth R Volkmann [9] Christopher P Denton [15] Marialuisa Bocchino [19] Kristin B Highland [17] Anna-Maria Hoffmann-Vold [41] Khanna D [46] Roth MD [47]
	Pulmonary Function Tests (PFTs)	Functional evaluation of lung capacity and gas exchange	↓ FVC (restrictive pattern), ↓ DLCO (diffusion impairment)	X Rebecca Sheng [9] Christopher P Denton [15] Abeer Ghuman [24]
Markers supporting the assessment of disease activity and prognosis	Metabolomics	Identification of early biomarkers of ILD presence and progression	L-leucine, isoleucine, xanthosine, AMP	Chantal Florence Meier [3]
	CII (Computer-Integrated Index)	Quantitative assessment of HRCT findings, even without clinical symptoms	Based on MLA, skewness, and kurtosis; correlates with DLCO and inflammation markers	Marialuisa Bocchino [19]
	Inflammatory and Immunological Biomarkers	Evaluation of disease activity and fibrotic/inflammatory profile	IL-6, CRP, CRPM, SP-D, CCL18, periostin, COMP, Pro-C3	X Rebecca Sheng [8] Abeer Ghuman [24]
	Autoantibody Profiling	Phenotype stratification and prognosis	ATA, ACA, anti-RNAP III, U3-RNP—associated with ILD risk and organ complications	Svetlana I Nihtyanova [23] Abeer Ghuman [24]

HRCT—high-resolution computed tomography, PFTs—pulmonary function tests, FVC—forced vital capacity, DLCO—diffusing capacity of the lung for carbon monoxide, NSIP—nonspecific interstitial pneumonia, UIP—usual interstitial pneumonia, ILD—interstitial lung disease, CII—computer-integrated index, MLA—mean lung attenuation, IL-6—Interleukin 6, CRP—C-reactive protein, CRPM—C-reactive protein metabolite, SP-D—surfactant protein D, CCL18—C-C motif chemokine ligand 18, COMP—cartilage oligomeric matrix protein, Pro-C3—propeptide of type III collagen, ATA—anti-topoisomerase I antibody, ACA—anti-centromere antibody, anti-RNAP III—anti–RNA polymerase III antibody, U3-RNP—U3 ribonucleoprotein antibody, ↓—decrise.

**Table 3:** Summary of pharmacological treatments for systemic sclerosis-associated interstitial lung disease (SSc-ILD).

Drug	Mechanism/Characteristics	Studies/Authors	Key Treatment Outcomes	Side Effects and Notes	References
Nintedanib	Tyrosine kinase inhibitor, inhibits processes leading to lung fibrosis and inflammation, affects fibroblast proliferation and ECM deposition	Distler et al. (SENSCIS), Khanna et al., Seibold JR, Kuwana M., Volkmann ER, Lui JK, Azuma A., Denton CP, Maher TM, Highland KB	Significantly slows FVC decline (e.g., 41 mL/year in SENSCIS); more effective in patients with rapid progression; can be safely combined with MMF	Most common side effect: diarrhea (75.7%); higher serious adverse events vs. placebo (16.6% vs. 8.7%)	[6,9,10,11,12,13,14,15,16,17,48,49,50,51,52,53,54]
Cyclophosphamide (CYC)	Immunomodulatory drug used in SSc with lung involvement, available IV and oral, inhibits inflammation and disease progression	Lui JK, Roth et al., Khanna et al., Bruni et al., Volkmann ER, Tashkin et al.	Beneficial effect on FVC%, especially in severe disease; no significant difference between IV and oral forms; benefits mainly within first year	Frequent side effects: leukopenia, anemia, hematuria; serious adverse events including deaths (11%) linked to age and comorbiditie	[6,20,25,46,47,55,56]
Mycophenolate mofetil (MMF)	Immunosuppressant that slows disease progression and improves lung function, often used combined with nintedanib	Lui JK, Highland KB, Sampaio-Barros PD et al.	Positive impact on FVC, especially in early/moderate stages; combination with nintedanib is safe; reduces vaccine responses (e.g., COVID-19 vaccines)	Side effects: diarrhea, nausea, skin ulcers; serious adverse events less common	[6,17,18]
Tocilizumab	Monoclonal antibody blocking IL-6 receptor, immunomodulatory, may slow early lung fibrosis progression	Sheng XR et al., Roofeh D. et al., Hoffmann-Vold AM	Slows FVC decline (e.g., −0.6% vs −4.0% placebo); potentially beneficial in early disease stage	Good safety profile, further studies needed, no effect on TGF-β and some inflammatory pathways	[7,8,41]
Rituximab (RTX)	Anti-CD20 monoclonal antibody; selective depletion of B lymphocytes, minimal effect on T cells	Goswami RP et al.; Maher et al.; Ebata S et al.; Roofeh D. et al.; Volkmann et al.; Ahmed et al.	Meta-analysis: FVC +4.5% (6 mo), +7.0% (12 mo); DLCO +3.5% and +4.1%; reduction in mRSS. RECITAL: similar FVC improvement as CYC at 24 weeks, no superiority of RTX. DESIRES: sustained mRSS improvement after additional 24 weeks; both early RTX and delayed RTX (after placebo) groups showed significant skin score reduction. Roofeh: improvement in FVC and mRSS vs control (12 mo). Volkmann: stabilization of lung function (2 yrs). Ahmed: skin thickness reduction and lung function stabilization in early diffuse SSc.	Better tolerated than CYC; lower incidence of adverse events (esp. infections); most common were infusion reactions; serious infections rare; in DESIRES, no deaths occurred. Larger multicenter studies still needed.	[29,30,31,33,43,44,58]
Pirfenidone	Antifibrotic agent; targets fibrotic pathways to slow disease progression	Tashkin DP et al.	Combination (Pirfenidone + MMF) accelerated FVC improvement within 6 months; improved quality of life. After 18 months, differences vs. MMF alone were less pronounced.	Higher rate of adverse events, mostly gastrointestinal (mild to moderate). Long-term safety and efficacy still under investigation.	[45]
Riociguat	Soluble guanylate cyclase stimulator with hypotensive, anti-inflammatory, and antifibrotic effects (animal studies)	Khanna D et al.	Slows FVC decline (2.7% vs. 7.6% placebo); potential use in ILD patients	Common side effects: nausea, diarrhea, headaches, peripheral edema; further clinical research needed	[26]

SSc-ILD—systemic sclerosis-associated interstitial lung disease, FVC—forced vital capacity, ECM—extracellular matrix, MMF—mycophenolate mofetil, CYC—cyclophosphamide, IL-6—Interleukin-6, TGF-β—transforming growth factor beta, IV—intravenous, COVID-19—coronavirus disease 2019, SENSCIS—systemic sclerosis–associated interstitial lung disease trial with nintedani, FDA—Food and Drug Administration.

## Data Availability

Not applicable.

## Abbreviations

The following abbreviations are used in this manuscript:

SSc	Systemic Scleroderma
ILD	Interstitial Lung Disease
PAH	Pulmonary Arterial Hypertension
ATA	Anti-Topoisomerase Antibodies
FVC	Forced Vital Capacity
CYC	Cyclophosphamide
MMF	Mycophenolate Mocofetil
mRSS	Modified Rodnan Scale
HCRT	High-Resolution Computer Tomography
SGRQ	St. George’s Respiratory Questionnaire
CRP	C-Reactive Protein
CRPM	C-Reactive Protein Metabolite (CRPM)
IL-6	Interleukin 6
DMARDs	Disease-Modifying Antirheumatic Drugs
PFTs	Pulmonary Function Tests
DLCO	Diffusing Capacity of the Lung for Carbon Monoxide
NSIP	Nonspecific Interstitial Pneumonia
UIP	Usual Interstitial Pneumonia
CII	Computer-Integrated Index
MLA	Mean Lung Attenuation
SP-D	Surfactant Protein D
CCL18	C Motif Chemokine Ligand 18
COMP	Cartilage Oligomeric Matrix Protein
Pro-C3	Propeptide of Type III Collagen
ATA	Anti-Topoisomerase I Antibody
ACA	Anti-Centromere Antibody
anti-RNAP III	Anti–RNA Polymerase III Antibody
U3-RNP	U3 Ribonucleoprotein Antibody
RTX	Rituximab
